# Comparison of Aqueous Depth Changes Following Cataract Surgery in Vitrectomized and Non-Vitrectomized Fellow Eyes

**DOI:** 10.3390/diagnostics15111429

**Published:** 2025-06-04

**Authors:** Mercè Guarro, Laura Sararols, Elena López, Meritxell Vázquez, Sergi Ruiz, Marc Biarnés

**Affiliations:** 1 Oftalmologia Mèdica i Quirúrgica Research, c/Tamarit 39, 08905 Sabadell, Barcelona, Spain; 2 Department of Ophthalmology, Hospital General de Granollers, Avda Francesc Ribas s/n, 08402 Granollers, Barcelona, Spain

**Keywords:** anterior chamber depth, aqueous depth, effective lens position, phacoemulsification, phacovitrectomy

## Abstract

**Background/Objectives**: The role of the vitreous in the effective lens position (ELP) is controversial in patients undergoing phacovitrectomy. The aim of this study was to compare the change in aqueous depth (AD), a surrogate of the ELP, in non-vitrectomized and vitrectomized fellow eyes. **Methods**: Post-hoc analysis of a prospective study conducted in OMIQ facilities (Barcelona, Spain) between 2021 and 2023. Patients with bilateral cataracts and a unilateral grade 2/3 epiretinal membrane underwent phacoemulsification in one eye and phacovitrectomy without endotamponade in the fellow eye. All eyes were implanted with an extended depth-of-focus intraocular lens after power calculation using the same biometer, technicians, formula, and surgeon. We compared the change in AD (mm and percentage) from baseline, and the role of vitrectomy without endotamponade on AD with a mixed-effects models. **Results**: We included 40 eyes (20 patients) with a mean age of 71.6 years, with 55% females. The mean change in AD was +1.51 (vitrectomized) and +1.42 mm (non-vitrectomized eyes), *p* = 0.33. The percent of change in AD was not different between groups (*p* ≥ 0.38) and phacovitrectomy had no effect on the change in AD on mixed-effects models (*p* > 0.10). **Conclusions**: The absence of the vitreous had a minimal influence on AD in these patients undergoing standard phacoemulsification or phacovitrectomy.

## 1. Introduction

Phacovitrectomy (combined phacoemulsification with intraocular lens [IOL] implantation and pars plana vitrectomy [PPV]) is increasingly used to treat patients aged 50 years or more with vitreoretinal disorders [1,2]. It provides faster visual recovery and makes a subsequent phacoemulsification, which is required in 100% of cases in some series [3], unnecessary.

After standard phacoemulsification multifocality is increasingly demanded by patients, who aim not only at emmetropia but also at presbyopia compensation. Nonetheless, current-generation formulas for IOL power calculation do not take into consideration the status of the vitreous (the recently released artificial intelligence-based formula LISA-PPV [4] aims to determine IOL power in previously vitrectomized eyes, but not in those undergoing prospective combined vitrectomy and phacoemulsification). The absence of the vitreous body can have an influence on postoperative effective lens position (ELP), which in turn may result in undesirable residual refractive errors after phacovitrectomy. Some studies have found a myopic shift [5,6,7,8], whereas others reported a hyperopic residual error [9] after this procedure. Comparison of vitrectomized and non-vitrectomized eyes from different patients [10]; heterogeneous indications for phacovitrectomy [5,9]; and different biometers, formulas, types of IOL, surgeons or gas tamponades [5] may be responsible for these seemingly contradictory results.

We compared the change in aqueous depth (AD), the distance between the corneal endothelium and the IOL anterior surface (a proxy for the ELP), between vitrectomized and non-vitrectomized fellow eyes of patients undergoing sequential phacovitrectomy in one eye and phacoemulsification in the other implanted with the same IOL type. The purpose was to determine the role of the absence of the vitreous on the ELP.

## 2. Materials and Methods

### 2.1. Study Design

This is a post hoc analysis of a pilot prospective, interventional, descriptive study aimed at determining visual performance in patients undergoing phacoemulsification in one eye and phacovitrectomy in the fellow eye due to a stage 2 or 3 epiretinal membrane [11] implanted with an extended-depth of focus AcrySof IQ Vivity (Alcon, Fort Worth, TX, USA) IOL, as seen in the Vivity MEM study. The study was conducted at OMIQ Research—Oftalmologia Mèdica i Quirúrgica facilities (an ophthalmology institute in Barcelona, Spain) between October 2021 and September 2023. The study adhered to the Tenets of the Declaration of Helsinki, was approved by the QuirónSalud-Catalunya Ethics Committee and all patients signed an informed consent form.

### 2.2. Patient Eligibility

Patients of any sex could be included in the Vivity MEM study if they were 18 years or older and had bilateral cataracts (any type: nuclear, cortical, posterior subcapsular, etc.), a grade 2 or 3 idiopathic epiretinal membrane [11] requiring PPV in one eye, and potential for post-surgical distance-corrected visual acuity of 20/32 or better.

Exclusion criteria included other visual comorbidities that could limit visual recovery in either eye (i.e., age-related macular degeneration, severe dry eye, corneal disease, etc.); a spherical equivalent ≤ −5.00 D; amblyopia; prior history of ocular trauma, intraocular or refractive surgery; and systemic conditions or medications that could affect visual outcomes. Additionally, for this post hoc study, eyes undergoing a neodymium-yttrium aluminum garnet (Nd:YAG) laser capsulotomy due to a posterior capsule opacification between baseline and final (6-month) visits were also excluded.

### 2.3. Methods

As part of the Vivity MEM study, all patients underwent a complete ophthalmic exam, which included monocular and binocular, corrected and uncorrected distance, intermediate and near visual acuity tests using Early Treatment Diabetic Retinopathy Study charts; contrast sensitivity tests using Pelli-Robson charts; dysphotopsias tests using the Light Distorsion Analyzer (Minho, Portugal); intraocular pressure tests with a Goldmann contact tonometer; anterior and posterior segment examinations; and macular and peripapilar spectral domain optical coherence tomography (OCT) tests with Spectralis HRA+OCT (Heidelberg Engineering, Heidelberg, Germany).

For this study, baseline (pre-surgery) and final (at least 6 months post-surgery) biometries were used to determine AD and anterior chamber depth (ACD, the distance between the corneal epithelium and the anterior IOL surface). These exams were conducted by experienced optometrists (MV, SR or EL) using the Lenstar LS 900 (Haag-Strait, Bern, Switzerland) biometer. Manual adjustments for proper positioning of peak signals derived from ocular structures were made preserving masking to eye status (vitrectomized or non-vitrectomized). In all eyes, the target refraction was emmetropia. The Barret Universal II formula was used with the AcrySof IQ Vivity IOL (Alcon, Fort Worth, TX, USA) constant recommended by the manufacturer.

All cases were operated on by the same surgeon (LS), with one month between each eye. Standard phacoemulsification was performed in the eye with the cataract using the Centurion system (Alcon Healthcare, Fort Worth, TX, USA), with a 2.4 mm incision at 180°, 5 mm capsulorrhexis and in-the-bag IOL implantation. For toric IOL, the Verion digital guided system (Alcon Healthcare, USA) was used for axis alignment. For the eye with the ERM, phacovitrectomy was performed using the Constellation system (Alcon Healthcare, USA). In these eyes, standard phacoemulsification was followed by PPV, with insertion of 25G trocars after cataract surgery, core vitrectomy, and moderate vitreous base shaving. Staining with Twin (Alchimia Srl, Padova, Italy) and dissection of the ERM and the internal limiting membrane was performed in all cases. No endotamponade was used in any case.

### 2.4. Statistical Analysis

We described the sample using the mean (standard deviation) for quantitative and *n* (percentage) for categorical variables. After checking the normality of the distribution of all quantitative variables, differences between groups (vitrectomized and non-vitrectomized eyes) were evaluated using the *t*-test.

We compared the difference in AD pre- and post-surgery between vitrectomized and non-vitrectomized eyes using the *t*-test, and the proportion of change in AD (expressed as (AD post-surgery − AD pre-surgery/AD pre-surgery) using the z-test. To evaluate the role of phacovitrectomy on the outcome (AD changes between pre- and post-surgery), we used multilevel mixed-models (adjusted for baseline ACD/AD and axial length) nested by patient, which also allow one to take into consideration the correlation between eyes of a given patient. All these analyses were repeated with ACD as the outcome.

We determined the sample size required to detect a clinically relevant difference of 0.50 D (the minimal step between IOL powers) between the control (phacoemulsification) and experimental (phacovitrectomy) groups in two independent samples, assuming a mean final refraction in the control group of 0.00 D (standard deviation 0.50 D), with a two-sided α = 0.05 and an 80% power.

All statistical analyses were conducted using Stata IC, version 15.1 (StataCorp, College Station, TX, USA). A two-tailed *p*-value < 0.05 was considered statistically significant and no adjustments for multiple comparisons were made [12].

## 3. Results

We included 20 of the 22 patients (40 eyes) included in the Vivity MEM study with a phacovitrectomy in one eye (due to the presence of an ERM) and a phacoemulsification in the fellow eye. In the two missing patients, the post-surgery biometry was not obtained. The minimal sample size needed to detect a difference of at least 0.50 D between groups was 17 patients (34 eyes). Table 1 shows the comparison of baseline characteristics between these groups in all participants. There were no statistically significant differences between groups in any of the variables considered.

**Figure 1:**
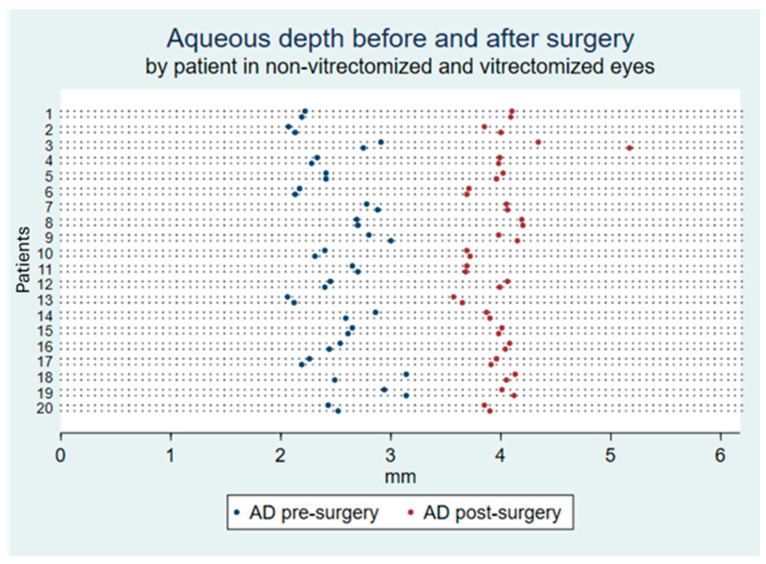
The dot chart shows AD before and after surgery in every eye of every participant (*y*-axis) stratified by non-vitrectomized (first) and vitrectomized (second) eyes. In all eyes there was an increase in AD after surgery. AD: aqueous depth.

The difference between groups in AD and ACD in pre- vs. post-surgery is shown in Table 2 and Figure 1. The results show that, although the mean change in AD and ACD is numerically larger for vitrectomized than non-vitrectomized eyes (i.e., the lens is more posteriorly located), the differences are very small and not statistically significant for any measurement or metric (*p* ≥ 0.31). Additionally, the percentage of eyes within +/−0.50 D and the mean post-operative spherical equivalent were not different between vitrectomized and non-vitrectomized eyes (95% vs. 95%, *p* = 1.00; and −0.09 D vs. −0.15 D, *p* = 0.37, respectively).

Table 3 shows the role of vitrectomy on AD and ACD change adjusted for potential confounders (baseline AD or ACD, and axial length). As compared to non-vitrectomized eyes, those vitrectomized showed a slight increase in AD and ACD change after surgery, but the results did not reach statistical significance (*p* = 0.11 and *p* = 0.10, respectively). On the other hand, a larger baseline AD or ACD was associated with a decreased change in AD or ACD after surgery, irrespective of the absence of vitreous and axial length.

## 4. Discussion

The results of the present study showed that the changes in AD and ACD, surrogate measurements for the ELP, were similar between vitrectomized and non-vitrectomized eyes 6 months after surgery. These results were consistent regardless of the used metric (difference in measurements in mm or in percentage of change) and analytical approach (comparison of means or mixed-models adjusted for potential confounders).

These results suggest that the absence of the vitreous has a small effect on the ELP. This is important, since 35% of the residual refractive error after cataract surgery is attributable to errors in estimating the postoperative ELP [13]. Given that the ELP is not provided in most current formulas, we used the AD or ACD as a surrogate for IOL position [5,9]. Others compared differences on residual refractive error between vitrectomized and non-vitrectomized eyes to evaluate the added effect of PPV [6,7]. In our study, the mean residual spherical equivalent and the percentage of eyes within +/−0.50 D were not statistically different between these two groups 6 months after surgery (*p* ≥ 0.37). These combined results provide further confirmation of the small influence PPV had on accurate preoperative IOL power determination in the current study.

Nonetheless, most previous studies report a myopic shift of up to −0.50 D in vitrectomized as compared to non-vitrectomized eyes [8,10,14]. Hamoudi et al. reviewed potential reasons for these findings [7]. One of them was a change in refractive index (*n*) in the vitreous cavity caused by replacement of vitreous gel by balanced salt solution after surgery [7]. We evaluated the results 6 months after surgery in cases where no endotamponade was used, and therefore the vitreous cavity was filled with aqueous humor (*n* = 1.336), with a refraction index very similar to that of the original vitreous (*n* = 1.337). Previous studies with a short follow-up could have been biased by the presence of balanced salt solution (*n* = 1.333) [15] in the vitreous cavity, the refractive index of which may be susceptible to manufacturer specifications, storing conditions, etc. However, everything else being equal, the lower refractive index of balanced salt solution should lead to a hyperopic shift. Other proposed explanations for myopization [7] included increases in axial length [10] or macular thickness [14]. We used the Lenstar LS 900 biometer (Haag-Streit AG, Bern, Switzerland), which is based on optical low-coherence reflectometry [16] and measures axial length from the corneal apex to the retinal pigment epithelium. As such, any previous macular thickening induced by the ERM could not affect our preoperative axial length measurements, precluding a postoperative myopic residual spherical equivalent. Shiraki et al. [5] further attributed the myopization to a forward positioning of the IOL in vitrectomized eyes even after the gas disappeared, which the authors argued may have been influenced by heterogeneous vitreous base shaving causing zonular laxity in some patients. We did not find a more anterior displacement of the IOL in vitrectomized eyes; the longer follow-up in our study (6 months vs. 1 month) and moderate vitreous base shaving (as opposed to complete removal) may explain these differences.

Other studies have reported a hyperopic shift or increases in ACD in vitrectomized eyes [9]. Indeed, the change in AD and ACD was slightly larger in vitrectomized than in non-vitrectomized eyes (+0.09 mm), which would be theoretically induce less than a +0.15 D change in final refraction [17]. This suggests that the absence of the vitreous offers less resistance to the capsular bag, allowing it to stabilize more posteriorly. A larger sample size would be required to test this trend, but in our study the difference in displacement was very small and not clinically significant, according to the similar postoperative spherical refraction in both groups. An exception would apply to cases requiring a high power IOL in a high hyperope, in whom slight changes in IOL position may affect the refractive outcome. Simulations conducted in an optical bench and real-world data derived from clinical studies in this subgroup are needed.

Additionally, other authors have reported no differences in post-surgical residual refraction between eyes that underwent phacovitrectomy and those that underwent phacoemulsification. Shiraki et al. [5] did not find statistically significant differences in median absolute error in the subgroup of eyes undergoing cataract surgery (0.31 D) and those undergoing phacovitrectomy without gas tamponade (0.20 D). Similarly, Mayer-Xanthaki et al. [18] did not find differences in refraction or ACD between these two groups using different formulas, nor did Chatzmichail et al. [19] in a recent large retrospective study. All these studies suggest that if no endotamponade is used, refractive changes are minimal. In this scenario, there are no differences in anterior segment structures (anterior chamber width, depth, and iridocorneal angle) using an anterior segment OCT [20], while other studies found anterior displacement of the ELP after combined surgery [21]. A summary of previous studies is shown in Table 4.

The limitations of this study include a relatively small sample size and the inability to directly measure the ELP. We used a surrogate variable [31] (the AD/ACD), which may not fully capture the true position of the IOL. Additionally, the results may not apply to other cases, including the implantation of the IOL in the sulcus [32], use of endotamponades [33], extensive vitrectomy of the vitreous base [34], high axial lengths [35], or in those with a history of episcleral surgery [36]. Phacovitrectomy with endotamponade in eyes with rhegmatogenous retinal detachment or indications requiring extensive vitreous base shaving can cause zonular damage and secondary bag instability [37,38,39].

In summary, in patients undergoing phacovitrectomy without endotamponade and extensive vitreous base removal, the absence of the vitreous did not have a significant impact on the estimated ELP. From a clinical standpoint, these results suggest that refractive surprises after phacovitrectomy without endotamponade are likely minimal. Further studies in other situations (use of endotamponade, longer follow-up, high hyperopes) are required to establish the influence of the vitreous on ELP and refractive outcomes.

## Figures and Tables

**Table 1:** Pre-surgical characteristics of study participants. Values represent mean (standard deviation) for continuous and *n* (percentage) for categorical variables. ACD: anterior chamber depth; AD: aqueous depth; D: diopters; N/A: does not apply.

Characteristics	Vitrectomized	Non-Vitrectomized	*p*-Value
Sample size, eyes	20	20	N/A
Age, years	71.6 (5.8)		N/A
Sex, female	11 (55.0%)		N/A
Right eye	10 (50%)	10 (50%)	1.00
AD, mm	2.50 (0.30)	2.54 (0.31)	0.69
ACD, mm	3.04 (0.29)	3.08 (0.31)	0.67
Pachymetry, μm	540.6 (36.3)	542.6 (37.3)	0.86
K1, D	43.23 (1.62)	43.37 (1.58)	0.78
K2, D	43.96 (1.55)	44.11 (1.59)	0.76
Mean K, D	43.67 (1.50)	43.95 (1.62)	0.57
Lens thickness, mm	4.58 (0.38)	4.52 (0.42)	0.65
Axial length, mm	23.42 (0.73)	23.36 (0.73)	0.81
Spherical equivalent, D	+1.04 (1.69)	+1.01 (1.49)	0.94

**Table 2:** Comparison of changes in AD and ACD pre- and post-surgery in vitrectomized and non-vitrectomized eyes. Values represent mean (standard deviation) for continuous variables and percentage for change. ACD: anterior chamber depth; AD: aqueous depth.

Characteristics	Vitrectomized	Non-Vitrectomized	*p*-Value
AD change, mm	+1.51 (0.33)	+1.42 (0.26)	0.33
AD change, %	+62.1% (17.1)	+57.7% (16.4)	0.41
ACD change, mm	+1.52 (0.33)	+1.42 (0.26)	0.31
ACD change, %	+50.8% (13.5)	+47.2% (12.6)	0.38

**Table 3:** Mixed-models for the characteristics influencing change in aqueous depth (top) and anterior chamber depth (bottom). The non-vitrectomized eye was the reference in the comparison vs. the vitrectomized eye. ACD: anterior chamber depth; AD: aqueous depth; CI: confidence interval; SE: standard error; vitrec: vitrectomized. * Statistically significant.

Characteristics	Coefficient (β), SE	95% CI	*p*-Value
Dependent variable: AD			
Vitrec vs. non-vitrect	0.07 (0.05)	−0.02 to 0.16	0.11
Baseline AD, mm	−0.63 (0.14)	−0.90 to −0.36	<0.001 *
Axial length, mm	−0.07 (0.06)	−0.20 to 0.05	0.25
Dependent variable: ACD			
Vitrec vs. non-vitrect	0.08 (0.05)	−0.01 to 0.17	0.10
Baseline ACD, mm	−0.63 (0.14)	−0.91 to 0.36	<0.001 *
Axial length, mm	−0.07 (0.06)	−0.19 to 0.06	0.28

**Table 4:** Summary of the main results from previous studies comparing changes in refractive error or ACD in eyes that underwent phacovitrectomy vs. those that underwent phacoemulsification. ACD: anterior chamber depth; BSS: balanced salt solution; DR: diabetic retinopathy; ERM: epiretinal membrane; ME: mean refractive prediction error; MH: macular hole; N/R: not reported; PPV: pars plana vitrectomy; RE: refractive error; RD: retinal detachment; SE: spherical equivalent; VMT: vitreomacular traction; w: with; w/o: without; *: includes patients with tamponade.

Study	*N* Eyes	Major Indication	Study Design	Measurement Method	Results (Refractive Error or Change in ACD)
Shioya, J., et al., 1997 * [22]	36	MH	Case series	Ultrasound (Alpha 20/20, Storz, Tuttlingen, Germany)	−0.55 D
Senn, P., et al., 2000 [23]	26	DR, ERM, uveitis	Prospective case series	N/R	−0.18 D
Suzuki, Y., et al., 2000 [24]	206	DR, MH, RD	Case series	N/R	−0.05 D
Jeoung, J.W., et al., 2007 [8]	154	DR, ERM, MH	Prospective case series	Ultrasound (A-scan 820, Zeiss, Jena, Germany)	−0.06 D
Kovács, I., et al., 2007 [25]	12	ERM, DR	Prospective case series	Ultrasound (Ultrascan, Alcon)	−0.79 D
Patel, D., et al., 2007 [6]	40	MH	Retrospective case series	Ultrasound (EchoScan US-1800, Nidek, Gamagori, Japan)	−0.39 D
Byrne, S., et al., 2008 * [14]	87	DR, ERM, MH, RD, miscellaneous	Retrospective case series	Optical (IOL Master, Zeiss)	−0.65 D
Falkner-Radler, C.I., et al., 2008 * [10]	40	ERM, MH	Clinical trial	Optical (IOL Master, Zeiss)	−0.52 D (−0.20 D with gas)
Schweitzer, K.D., et al., 2008 * [26]	54	DR, ERM, MH	Consecutive case series	Optical (IOL Master, Zeiss)	+0.16 D (−0.30 D with gas)
Manvikar, S.R., et al., 2009 * [27]	59	DR, ERM, MH, RD	Retrospective case series	Optical (IOL Master, Zeiss)	−0.10 D (+0.03 D with gas)
Hwang, H.S., et al., 2011 * [28]	40	MH	Prospective case series	Ultrasound (Ecograph axis II, Quantel Medical, Clermont-Ferrand, France)	−0.61 D
Sun, H.J., et al., 2011 * [29]	23	ERM, MH	Retrospective case series	Ultrasound (A/B scan Workstation, Paradigm MI, Salt Lake City, UT, USA)	−0.46 D
Mijnsbrugge, J.V., et al., 2018 [9]	40	ERM, floaters, VMT	Prospective case series	Optical (IOL Master 700, Zeiss)	Phacoemulsification: change SE −0.05 D; change in ACD 1.99 mm Phacovitrectomy: change in SE −0.18 D (*p* = 0.18); change in ACD 2.12 mm (*p* = 0.044)
Shiraki, N., et al., 2020 * [5]	76	ERM, MH and RD	Retrospective case series	Optical (IOL Master 500, Zeiss)	Group A: phacoemulsification: +0.08 D Group B: phacovitrectomy w/o gas: −0.07 D Group C: phacovitrectomy w/gas: −0.82 D, *p* < 0.001 vs. A and B
Tranos, P.G., et al., 2020 * [30]	109	ERM, MH	Retrospective case series	Optical (Lenstar 9000, Haag-Streit, Köniz, Switzerland)	Phacovitrectomy: +0.59 D Phacoemulsification after PPV: +0.35 D, *p* = 0.01
Mayer-Xanthaki, C.G., et al., 2022 [18]	40	ERM	Prospective case series	Optical (IOL Master 700, Zeiss)	Phacoemulsification: change in ACD −0.10 mm Phacovitrectomy: change in ACD −0.12 mm, *p* = 0.36
Chatzimichail, E., et al., 2023 [19]	160	ERM, MH, RD	Retrospective case–control	Optical (IOL Master 700, Zeiss)	No difference in ACD between groups defined by endotamponade: BSS (4.60 mm), air (4.52 mm) or gas (4.56 mm; *p* = 0.40). The refractive prediction error was slightly higher in the gas than in the phacoemulsification group (*p* ≤ 0.012)
Crincoli, E., et al., 2024 [21]	219		Prospective case series	Biometry	Phacovitrectomy: −0.29 D Phacoemulsification: −0.03 D, *p* = 0.023

## Data Availability

Data may be available from the authors upon reasonable request.
